# The great escape: a *Shigella* effector unlocks the septin cage

**DOI:** 10.1038/s41467-024-48208-1

**Published:** 2024-05-15

**Authors:** Ana T. López-Jiménez, Gizem Özbaykal Güler, Serge Mostowy

**Affiliations:** Department of Infection Biology, London School of Hygiene and Tropical Medicine, London, UK

**Keywords:** Ubiquitylation, Cellular microbiology

## Abstract

*Shigella*, an important human pathogen, can secrete effector proteins to invade host cells and evade mechanisms of cell-autonomous immunity. In a new manuscript published in *Nature Communications*, Xian et al. report that the *Shigella* kinase effector OspG promotes the ubiquitination of septin cytoskeletal proteins to evade cage entrapment.

*Shigella* is a causative agent of dysentery responsible for over 200,000 deaths per year. For infection of human cells, *Shigella* employs a Type 3 Secretion System (T3SS) to deliver ∽30 effector proteins in a highly orchestrated manner. While early effectors promote host cell invasion and escape from the phagocytic vacuole to the cytosol, late effectors mediate evasion of host immune defenses, including mechanisms of cell-autonomous immunity. Cell-autonomous immunity is the ability of immune and non-immune cells to protect themselves against intracellular pathogens using evolutionarily conserved defense pathways^1^. As a human-adapted intracellular pathogen, *Shigella*’s ability to counteract cell-autonomous immunity has been the focus of intense investigation. However, our mechanistic understanding is limited as *Shigella* effectors often have pleiotropic roles and target host pathways in a multi-layered manner. Prominent examples of effectors important for *Shigella* virulence include (i) IcsB and VirA that mediate escape from antibacterial autophagy^2,3^, (ii) IpaH9.8 that counteracts guanylate-binding protein (GBP)-mediated immunity^4,5^, (iii) OspC3 that targets Caspase-4 and Caspase-11 to avoid inflammatory cell death^6,7^, and (iv) OspC1 and OspC3 that supress the expression of interferon-inducible immune genes^8^ (Fig. 1A).

**Fig. 1:**
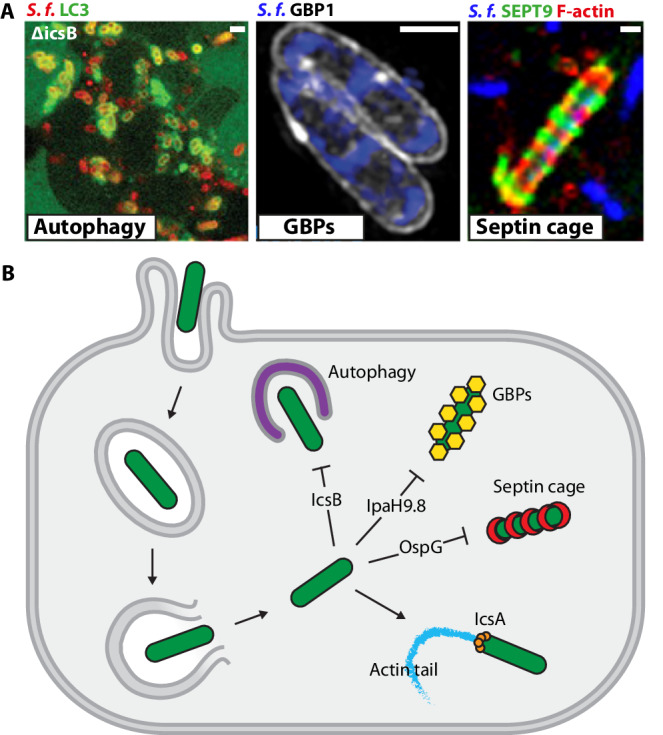
*Shigella* interactions with cell-autonomous immunity. **A** Fluorescent microscopy images of *Shigella flexneri* (*S.f*.) being targeted by antibacterial autophagy^2^ (left), GPB-mediated immunity^4^ (middle), and septin cage entrapment^11^ (right). Scale bars = 2, 1, and 1 μm, respectively. **B** Stages in the entry, vacuolar escape, and spread of *Shigella* by actin-based motility (IcsA). *Shigella* effectors that evade antibacterial autophagy (IcsB), GBP-mediated immunity (IpaH9.8), and septin cage entrapment (OspG) are highlighted.

Septins are a family of cytoskeletal proteins with key roles in cell division and host–pathogen interactions^9^. As compared to other cytoskeletal proteins, septin assembly is unconventional—different family members form hetero-oligomeric complexes, which assemble into filaments and interact with actin, microtubules, and membranes. Septin biology has been historically linked to posttranslational modifications (PTMs), although the precise role of PTMs in septin assembly and function is mostly unknown. Septins were among the first proteins reported to be modified by SUMOylation (a ubiquitin-like PTM), and work has shown roles for SUMOylation in septin filament bundling and cell division^10^. A role for the cytoskeleton in cell-autonomous immunity was discovered from studying septin–*Shigella* interactions, where cytosolic bacteria are compartmentalized by septin cage-like structures for targeting to antibacterial autophagy^11^. Over a decade of research performed in vitro using purified proteins, in cellulo using tissue culture cells, and in vivo using zebrafish infection models has shown that septin cages are antibacterial, preventing *Shigella* replication and dissemination^11–13^. However, why only a fraction of cytosolic bacteria are recognized by septin caging is poorly understood, and whether *Shigella* effectors can manipulate septin biology has been a long-standing question in the field. Reporting in *Nature Communications*, Xian et al. discover that a *Shigella* kinase effector OspG targets host ubiquitination pathways to inhibit septin assembly and evade cage entrapment^14^.

## A newly identified role for *Shigella* OspG to counteract septin-mediated cell-autonomous immunity

Ubiquitination is a well-studied PTM that plays a pivotal role in host–pathogen interactions. Ubiquitination is achieved by the sequential action of three sets of enzymes: ubiquitin-activating (E1), ubiquitin-conjugating (E2), and ubiquitin ligases (E3), that together catalyze a wide range of ubiquitin signals. Despite the vast repertoire of E1, E2, and E3 enzymes present in human cells, relatively few *Shigella* effectors have been found to target host ubiquitination pathways. In this case, work has shown that (i) IpaH1.4 and IpaH2.5 antagonize M1 ubiquitin chains by targeting the E3 ligase LUBAC to degradation^15,16^, and (ii) OspG and OspI bind E2 enzymes (including UbcH5b and UBC13, respectively) to downregulate host immune responses mediated by nuclear factor-κB (NF-κB)^17–19^.

For their study^14^, Xian et al. set out to investigate the kinase activity of OspG, whose precise molecular target had remained elusive for many years. The use of large-scale phosphoproteomics identified CAND1 (cullin-associated NEDD8-dissociated protein1), a key assembly factor of the E3 family of cullin-RING ubiquitin ligases (CRLs), as being phosphorylated by OspG. CAND1 was phosphorylated on several serine and tyrosine residues, indicating that OspG acts as an unusual dual serine/tyrosine kinase. Pull-down assays and liquid chromatography–mass spectrometry (LC-MS) showed that OspG-mediated phosphorylation of CAND1 regulates its interaction with several cullins, suggesting that its impact on host E3 ligase activity is widespread. Next, Tandem Ubiquitin Binding Entities (TUBE) pull down in cells was used to profile the impact of OspG on the host ubiquitome during *Shigella* infection and found several cellular pathways to be affected, including the NF-κB pathway. Strikingly, multiple septin family members (SEPT2, SEPT7, SEPT9, SEPT10, and SEPT11) presented higher levels of ubiquitination in cells infected by *Shigella* in the presence of OspG. Immunoprecipitation experiments with flag-tagged septins co-expressed with OspG confirmed robust ubiquitination of SEPT6, SEPT9, and SEPT10; weaker ubiquitination was observed for SEPT2, SEPT7, and SEPT11. Taken together, these findings demonstrate that phosphorylation of CAND1 by OspG causes a global shift in substrate ubiquitination through CRLs, and in this way, the kinase activity of OspG promotes ubiquitination of multiple septin family members. Considering recent work showing that septin filaments based on hetero-octameric complexes (SEPT2-SEPT6-SEPT7-SEPT9-SEPT9-SEPT7-SEPT6-SEPT2) link actin stress fibers to membrane^20^, and the previously reported role for SEPT9 in *Shigella* cage entrapment^11^, these new data support the hypothesis that septin filaments associated with the bacterial surface are based on hetero-octameric complexes.

How does ubiquitination modify septin assembly and function? It is well known that different ubiquitin signals can preferentially direct substrates towards different cellular pathways. For example, K63-linked polyubiquitin chains target substrates to autophagic degradation, whereas K48-linked polyubiquitin chains target substrates to proteasome degradation. *Shigella*-septin caging has previously been associated with ubiquitination and antibacterial autophagy, where immunostaining experiments and super-resolution microscopy suggested that septins and K63-linked polyubiquitin chains form separate microdomains on the bacterial surface^11,21^. For their study^14^, Xian et al. show that ubiquitinated SEPT9 contains mixed K6-, K27-, and K33-linked polyubiquitin chains, suggesting non-canonical roles for ubiquitination in septin biology beyond protein degradation. Considering this, the authors tested if OspG-mediated ubiquitination regulates the interaction between different septin family members. Immunoprecipitation experiments revealed a reduced association of SEPT9 with SEPT6 and SEPT7 in the presence of OspG expression, demonstrating that septin hetero-oligomeric complex formation and higher-order assembly are partially impaired upon ubiquitination. In agreement, the authors used quantitative microscopy and observed significantly more *Shigella* septin cages (~25%) in cells infected with an *ospG*-deletion mutant as compared to cells infected with OspG-expressing bacteria (~15%). It is worth noting that the specific construction of the *ospG* deletion mutant is important since a *mxiE*-deletion mutant (*mxiE* encodes a transcription factor responsible for upregulation of late effectors, including OspG) failed to promote septin cage entrapment. Immunostaining infected cells with FK2 (an antibody shown to recognize both mono- and polyubiquitinated proteins) showed that ubiquitin is recruited to SEPT9 and SEPT7 cages that entrap bacteria expressing OspG, supporting a role for OspG in septin ubiquitination. From these observations, the authors conclude that OspG promotes ubiquitin-mediated deconstruction of the *Shigella*-septin cage.

## Perspectives

In summary, Xian et al. have discovered a mechanism evolved by *Shigella* to counteract cell-autonomous immunity by ubiquitinating septins in an OspG-dependent manner (Fig. 1B). This discovery is exciting for both infection and cytoskeleton biology, highlighting a fundamental role for non-canonical ubiquitin linkages in the prevention of septin assembly. It is next of great interest to investigate the mechanisms and structural details underlying the ability of ubiquitin to prevent hetero-oligomeric complex formation and higher-order septin assembly. More broadly, Xian et al. provide insights into the manipulation of host ubiquitination pathways by *Shigella* and expand the breadth of cellular processes regulated by ubiquitination. Considering this newly identified kinase-dependent role for OspG, the phosphorylation of CAND1 to manipulate CRL complex activity may emerge as a common regulatory mechanism in eukaryotic cells. In the future, interdisciplinary approaches involving bacterial infection (using *Shigella* and other intracellular pathogens), ‘omics technologies, and high-throughput/high-content screening are likely to reveal further roles for PTMs in septin biology and cellular homeostasis^22^.

Beyond evasion of septin cage entrapment, what are the downstream consequences of OspG-mediated ubiquitination on host defense? Considering that OspG globally modifies the host ubiquitome, it is important to determine the multiple roles of OspG in the regulation of cellular and immune processes in addition to septin ubiquitination. Although Xian et al. do not perform survival assays or in vivo work to test for virulence of bacteria lacking *ospG*, it is well known that investigation of septin biology at the cellular and whole animal level is highly complex due to their diverse roles in cellular homeostasis. To address this, single-cell analysis focusing on *Shigella* septin cages undergoing OspG-mediated deconstruction may be required to fully characterize the impact of ubiquitination on entrapped bacteria. Studying how septin assembly and ubiquitination are coordinated with each other, and also with other cellular processes (such as antibacterial autophagy and GBP recruitment), will be necessary for a complete understanding of cell-autonomous immunity. As we better understand these cellular processes, we can aim to develop therapies that target them for enhanced infection control.
